# Signet‐ring cell large B‐cell lymphoma: A potential diagnostic pitfall with signet‐ring cell carcinoma

**DOI:** 10.1002/ccr3.2958

**Published:** 2020-06-03

**Authors:** Vijay Patel, Sergio Pina‐Oviedo

**Affiliations:** ^1^ Department of Pathology University of Arkansas for Medical Sciences Little Rock Arkansas USA

**Keywords:** differential diagnosis, lymphoma, morphology, pitfall, signet‐ring, variant

## Abstract

This study reveals the importance of recognizing uncommon histologic variants in diffuse large B‐cell lymphoma, such as the signet‐ring cell variant, which may result in an erroneous or delayed diagnosis with potential impact in patient treatment.

## Graphical abstract

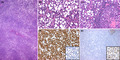

## DEAR EDITOR

**Figure 1:**
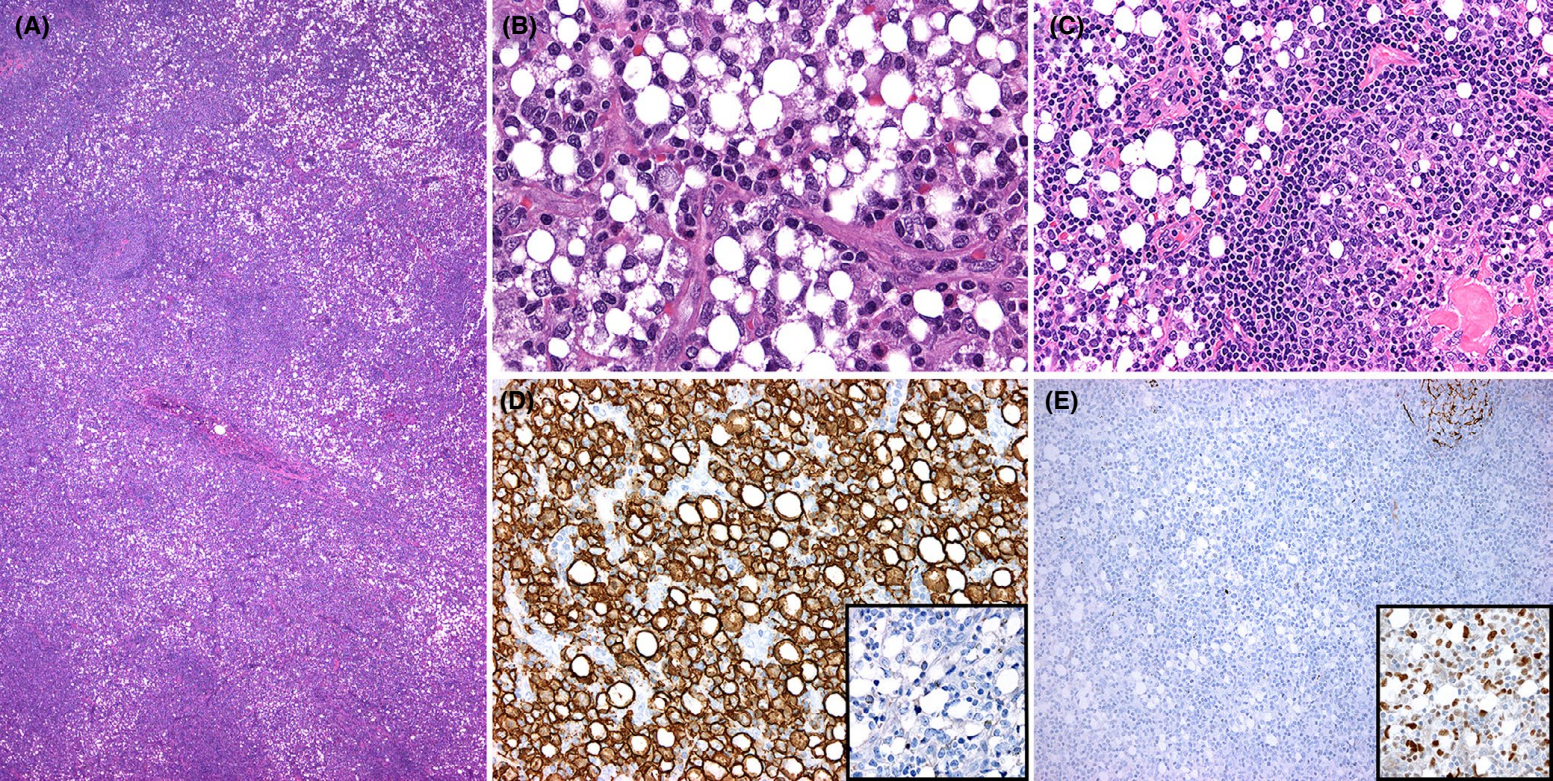
A, Effacement of the lymph node architecture by an atypical clear cellular infiltrate with few residual lymphoid follicles. B, The diffuse areas are composed of sheets of signet‐ring cells. C) Neoplastic lymphoid follicle with >15 centroblasts, few centrocytes, and occasional signet‐ring cells. D, Immunohistochemical stains show that the signet‐ring cells are positive for CD20 and negative for pan‐cytokeratin (D, inset). E, CD21 demonstrates lack of follicular dendritic cell meshworks in diffuse areas and few residual lymphoid follicles (top, right). The signet‐ring cells are positive for bcl‐6 (E, inset)

A 56‐year‐old man presented with a 4‐cm left inguinal lymphadenopathy. Lymph node excision showed effacement of the architecture by an atypical infiltrate with few residual lymphoid follicles (Figure 1A). The infiltrate consisted of signet‐ring cells and cells with multivacuolated cytoplasm (Figure 1B). The lymphoid follicles lacked polarization and contained signet‐ring cells and >15 centroblasts per follicle (Figure 1C). By immunohistochemistry, the signet‐ring cells were positive for CD20 (Figure 1D) and negative for pan‐cytokeratin (Figure 1D, inset). CD21 highlighted follicular dendritic cell meshworks in lymphoid follicles but lack of them in diffuse areas (Figure 1E). The signet‐ring cells were also positive for bcl‐6 (Figure 1E, inset), bcl‐2, CD10, and MUM1, and negative for T‐cell markers. Ki‐67 was 40%. Flow cytometry detected a CD10+ B‐cell population with variable forward scatter and lack of surface light chains. Fluorescence in situ hybridization showed rearrangement of the *BCL6* gene. A diagnosis of diffuse large B‐cell lymphoma (DLBCL) with a minor component of follicular lymphoma was established. Signet‐ring cell DLBCL is rare, with <100 reported cases to date.^1^, ^2^ Although this morphology has no prognostic impact, its recognition is crucial to avoid a misdiagnosis of metastatic signet‐ring cell carcinoma to lymph node, leading to inappropriate management.^1^, ^2^

## CONFLICT OF INTEREST

None declared.

## AUTHOR CONTRIBUTIONS

VP and SPO: contributed equally to the preparation of this work.
